# A guide to writing systematic reviews of rare disease treatments to generate FAIR-compliant datasets: building a Treatabolome

**DOI:** 10.1186/s13023-020-01493-7

**Published:** 2020-08-12

**Authors:** Antonio Atalaia, Rachel Thompson, Alberto Corvo, Leigh Carmody, Davide Piscia, Leslie Matalonga, Alfons Macaya, Angela Lochmuller, Bertrand Fontaine, Birte Zurek, Carles Hernandez-Ferrer, Carola Rheinard, David Gómez-Andrés, Jean-François Desaphy, Katherine Schon, Katja Lohmann, Matthew J. Jennings, Matthis Synofzik, Olaf Riess, Rabah Ben Yaou, Teresinha Evangelista, Thiloka Ratnaike, Virginie Bros-Facer, Gulcin Gumus, Rita Horvath, Patrick Chinnery, Steven Laurie, Holm Graessner, Peter Robinson, Hanns Lochmuller, Sergi Beltran, Gisèle Bonne

**Affiliations:** 1 Sorbonne Universite - Inserm UMRS 974, Center of Research in Myology, Institut de Myologie, G.H. Pitié-Salpêtrière Paris, 47, boulevard de l’Hopital, F-75 651 Paris Cedex 13, France; 2 Children’s Hospital of Eastern Ontario Research Institute, Ottawa, Canada; 3 CNAG-CRG, Centre for Genomic Regulation (CRG), Barcelona Institute of Science and Technology (BIST), Barcelona, Spain; 4 The Jackson Laboratory For Genomic Medicine, Farmington, CT 06032 USA; 5 Paediatric Neurology, Vall d’Hebron University Hospital and VHIR (Euro-NMD, ERN-RND), 08035 Barcelona, Spain; 6 Department of Clinical Neurosciences, University of Cambridge School of Clinical Medicine, Cambridge Biomedical Campus, Cambridge, UK; 7 Institute of Medical Genetics and Applied Genomics, University of Tuebingen, Tübingen, Germany; 8 Department of Biomedical Sciences and Human Oncology, School of Medicine, University of Bari Aldo Moro, Bari, Italy; 9 Institute of Neurogenetics, University of Lübeck, 23538 Lübeck, Germany; 10 Department of Neurodegenerative Diseases, Hertie-Institute for Clinical Brain Research and Center of Neurology, University of Tübingen, Tübingen, Germany; 11 German Center for Neurodegenerative Diseases (DZNE), University of Tübingen, Tübingen, Germany; 12 Unité de Morphologie Neuromusculaire, Institut de Myologie, GHU Pitié-Salpêtrière, Paris, France; 13 Sorbonne Université, AP-HP, INSERM, Centre de référence Des Maladies Neuromusculaires Nord/Est, Ile de France, Paris, France; 14 Department of Paediatrics, Cambridge University Hospitals NHS Foundation Trust, Cambridge, England; 15 Department of Clinical Neurosciences, University of Cambridge, Cambridge, UK; 16 EURORDIS – Rare Diseases Europe, Paris, France; 17 Department of Clinical Neurosciences, School of Clinical Medicine, University of Cambridge and MRC Mitochondrial Biology Unit, Cambridge Biomedical Campus, Cambridge, UK; 18 Department of Neuropediatrics and Muscle Disorders, Medical Center - University of Freiburg, Faculty of Medicine, University of Freiburg, Freiburg im Breisgau, Germany; 19 Division of Neurology, Department of Medicine, The Ottawa Hospital, Ottawa, Canada; 20 Brain and Mind Research Institute, University of Ottawa, Ottawa, Canada

**Keywords:** Rare diseases, Systematic literature reviews, Treatment knowledge-base

## Abstract

**Background:**

Rare diseases are individually rare but globally affect around 6% of the population, and in over 70% of cases are genetically determined. Their rarity translates into a delayed diagnosis, with 25% of patients waiting 5 to 30 years for one. It is essential to raise awareness of patients and clinicians of existing gene and variant-specific therapeutics at the time of diagnosis to avoid that treatment delays add up to the diagnostic odyssey of rare diseases’ patients and their families.

**Aims:**

This paper aims to provide guidance and give detailed instructions on how to write homogeneous systematic reviews of rare diseases’ treatments in a manner that allows the capture of the results in a computer-accessible form. The published results need to comply with the FAIR guiding principles for scientific data management and stewardship to facilitate the extraction of datasets that are easily transposable into machine-actionable information. The ultimate purpose is the creation of a database of rare disease treatments (“Treatabolome”) at gene and variant levels as part of the H2020 research project Solve-RD.

**Results:**

Each systematic review follows a written protocol to address one or more rare diseases in which the authors are experts. The bibliographic search strategy requires detailed documentation to allow its replication. Data capture forms should be built to facilitate the filling of a data capture spreadsheet and to record the application of the inclusion and exclusion criteria to each search result. A PRISMA flowchart is required to provide an overview of the processes of search and selection of papers. A separate table condenses the data collected during the Systematic Review, appraised according to their level of evidence.

**Conclusions:**

This paper provides a template that includes the instructions for writing FAIR-compliant systematic reviews of rare diseases’ treatments that enables the assembly of a Treatabolome database that complement existing diagnostic and management support tools with treatment awareness data.

## Background

In Europe, rare diseases (RD) are defined as those that affect one in two thousand individuals or fewer. Globally they affect around 6% of the population, and this means that, collectively, healthcare providers deal with a considerable number of patients with a rare disease, of which over 70% are genetically determined [1, 2]. A recent calculation of the cumulative prevalence at a global level based on the Orphanet epidemiology data file estimates that, regarding the 2017 population, there is a minimum prevalence of 3.5–5.9%, which corresponds to 263–446 million persons affected worldwide [2]. The problem is that each of those patients has a condition that they share with only a small number of individuals locally and sometimes even globally. For this reason, it is difficult to ensure that patients can access the right medical expertise as doctors seldom see such cases, and this fact contributes to a delay in diagnosis. A survey has shown that for a representative sample of 8 rare diseases, 25% of the patients wait 5 to 30 years to have a diagnosis, and 40% receive a wrong diagnosis before the final one [3, 4]. Living with a rare disease is a challenge and the lack of diagnosis – either because one exists but has not been identified for a particular patient or because the patient has a “syndrome without a name” (SWAN) – is a cause of extreme stress for patients and their families. In 2016, in an effort to help those undergoing the “diagnostic odyssey”, a group of rare disease organisations issued recommendations to address the specific needs of undiagnosed rare disease patients [4]. These include recognition as a distinct population, support through national and international programmes in which patients and their advocates are directly involved, and ethical international data sharing that accelerates innovation and discovery. International consortia (UDNI, IRDiRC) aim towards the next steps and strategies finding a diagnosis also in patients who already had comprehensive genetic testing [5]. Clinical research in these patient populations is also a challenge, as it requires the specific features of the rare disease to be addressed and often requires patient numbers that cannot be obtained in one clinic or country [3]. Only international collaboration can shorten the diagnostic delay and gather the cohorts required for clinical research by enabling the sharing of resources and data in an ethical manner that protects patients’ confidential data while enabling research and pooling of expertise.

Another issue has to do with treatment delay. Although rare diseases treatments are still scarce, the existing ones are sometimes overlooked as a consequence of that rarity and its application may be delayed. This is particularly true for variant and mutation-specific treatments as the ones for Congenital Myasthenia Syndromes [6].

**Fig. 1:**
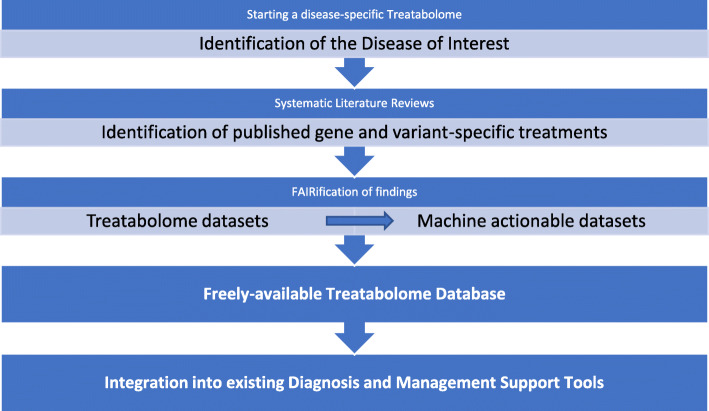
The Treatabolome is a project aiming at collecting and making freely available information about gene and variant-specific treatments for rare diseases. It starts by selecting a disease (or disease-group of interest) of interest and running a systematic literature review to identify the gene and variant-specific treatments that have been published. The resulting datasets will comply to the FAIR guiding principles of data stewardship to enable its easy translation to machine-readable data elements. The resulting database will be freely available online and will be made available for integration into existing diagnosis and management support tools, e.g. the GPAP platform of RD-Connect

In the current paper, we propose a common methodology for developing systematic literature reviews feeding into an innovative concept of a knowledge base of rare diseases’ treatments, as part of the European project Solve-RD. “Solve-RD - solving the unsolved rare diseases” is a research project funded under the European Union’s Horizon 2020 research and innovation programme (2018–2022). It echoes the ambitious goals set out by the International Rare Diseases Research Consortium (IRDiRC) to deliver by 2020 diagnostic tests for most rare diseases and 200 new therapies [the latter achieved 3 years ahead of schedule [7]], as the current diagnostic and subsequent therapeutic management of rare diseases is still highly unsatisfactory for a large proportion of patients – the unsolved RD cases. The concept behind Solve-RD is therefore to promote the diagnosis of cases by an integrated “beyond the exome” approach that combines Omics and generates a “genetic knowledge web” improving the diagnostic yield for rare disease patients. Solve-RD develops mechanisms for ensuring patients solved through this project are put on the precise path for appropriate therapy and further research. This aim will be served by the production of a publicly-available “Treatabolome” knowledge base to enable the flagging of treatable genes and variants to the sample-submitting clinician and at the same time identifying patient cohorts and bio-samples availability for clinical research. A substantial proportion of treatments that are already available for rare diseases are only suitable for patients with a particular genetic defect, as exemplified in a recent paper for Congenital Myasthenic Syndromes [6], and with the coming era of personalised medicine, this number is expected to grow. Enabling the flagging of treatable genes and variants to the clinician analysing the case means that the patient can be assessed for the relevant treatment the moment diagnosis is reached. The Treatabolome will thus enhance the visibility and accessibility of rare disease therapies (Fig. 1). This is needed as only 6% of the rare diseases have a known treatment at present [8] and clinicians are often not immediately aware of a gene or variant-specific treatment and can only discover this information after a relatively onerous search of the literature. The Treatabolome complements the omics approach to diagnosis by adding an innovative therapeutic information branch pointing to gene and variant-specific treatments.

**Fig. 2:**
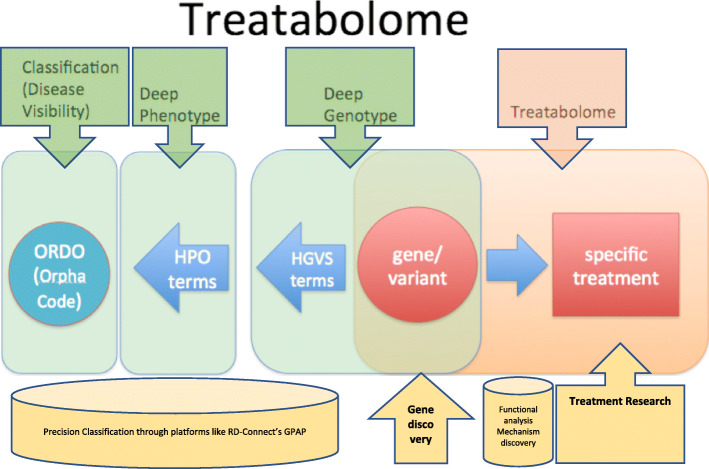
The Treatabolome is a precision medicine project. Departing from deep phenotyping using HPO terms it makes the correspondence with the causative gene variant designated according to HGVS convention, enabling a precise ORDO classification of the condition. If described, the Treatabolome will point the corresponding gene and variant-specific treatment. ORDO – Orphanet Rare Diseases Ontology. HPO – Human Phenotype Ontology. HGVS – Human Genome Variation Society. GPAP – Genome-Phenome Analysis Platform from RD-Connect

Our goal with the Treatabolome is to make this information more readily and immediately accessible. In order to be founded on the best evidence available, it must still begin with an analysis of the literature, but by producing a systematic review that gathers the information in a computer-accessible manner that can be used for the assembly of the Treatabolome database, we can reduce the work that clinicians must do to find the information in future. A Solve-RD task force is currently working towards building that database by working with disease experts to manually capture gene and variant information from the literature by means of a systematic literature review, mapping it to existing phenotype, gene and classification ontologies and correlating it to existing specific therapies in a database developed by computer experts (Fig. 2). Although previous systematic reviews have been written about rare diseases and genetic conditions, the singularity of the Treatabolome is that they are centred on gene and variant specific treatments and will translate into a precision medicine endeavour. The Treatabolome systematic reviews will share a common research question and are expert-led with direct involvement of specialists in each rare disease area, starting by experts from the four European Reference Networks [9] that participate in Solve-RD. However, our aim is to reach out to every ERN and invite experts from around the world to take the lead in their area of expertise.

It is not hard to anticipate that in the future the sustainability of this project will depend on a “big data to knowledge” (BD2K) methodology that uses text-mining of scientific publications for accruing and updating the knowledge database [10]. Therefore, the task force is working on a pilot of a text-mining tool to serve that purpose.

## Aims

The present paper aims to lay out the common ground and rules for all the Treatabolome expert-led systematic literature reviews (SLR) and incentivise the adoption of the FAIR-guiding principles for scientific data management and stewardship [11] as that later facilitate the translation of human-readable scientific papers into computer-actionable data. Under these FAIR principles, the data and metadata required for the translation of content, tools, algorithms, consents, and workflows described in the systematic reviews are collected into transparent, reproducible, and reusable digitally-accessible datasets. Based on the interpretation of metadata, digital devices can take appropriate action on approved areas without the need for direct human intervention, a state denominated “machine-actionability.” Such digital resources can autonomously determine how to act when presented with an unknown digital object as they are capable of determining the structure, reusability and intent of the object, through the interpretation of license, consent, and other data use constraints. These mechanisms enable the computer to “judge” if specific data can be shared, to give an example of machine actionable application.

The FAIR-compliant systematic reviews for rare diseases’ treatments will feed the Treatabolome database to enable expertise sharing. The aim is to increase treatment visibility by flagging existing rare diseases’ gene and variant-specific treatments each time a diagnosis is reached, through the incorporation of the Treatabolome datasets into diagnosis and management support tools.

## Methods

**Fig. 3:**
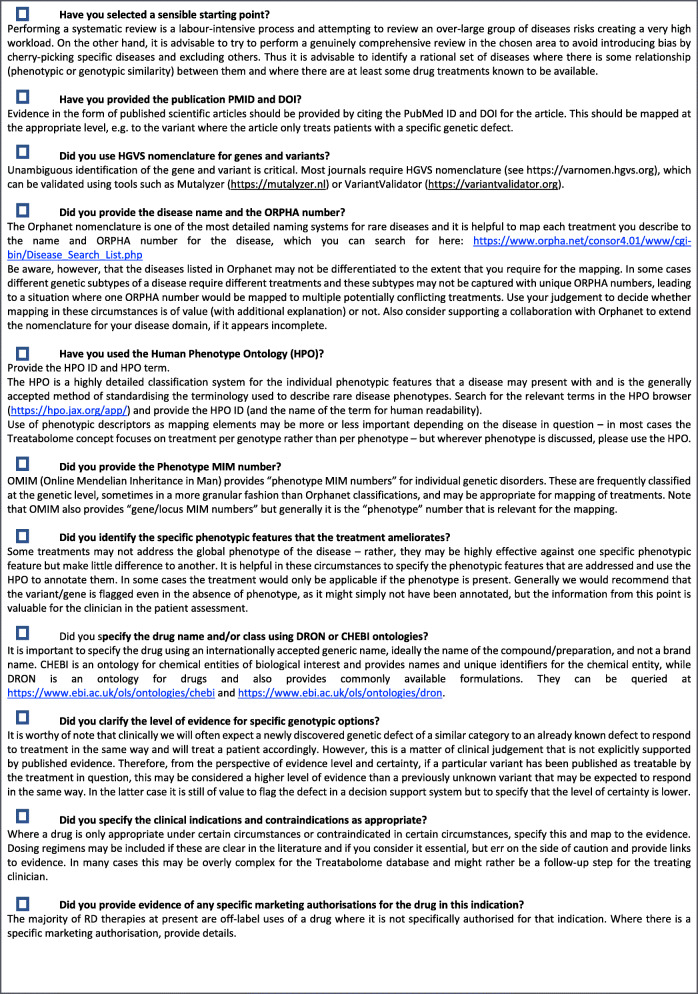
The Treatabolome Systematic Review Checklist

The starting point within the Treatabolome task is to engage clinical experts in the production of state-of-the-art systematic reviews of specific treatments for the rare diseases of their expertise. The critical addition to the workflow, in comparison with a traditional systematic review, is the capture of evidence linking treatment with specific genotype to enable the creation of a Treatabolome database flagging potentially treatable genes and variants. This information is mapped to different ontologies and classification systems. A checklist with recommendations has been composed to assist with the harmonisation of the different Treatabolome systematic reviews (Fig. 3). We describe below the proposed methodology for these SLRs, including an explanation of the checklist recommendations.

### Systematic review protocol

The systematic reviews should follow a written “Systematic Review Protocol” (Additional file 1) based on the PROSPERO template from the Centre for Reviews and Dissemination [12]. The advantage of using a PROSPERO-inspired protocol is that it was explicitly designed for Systematic Literary Reviews (SLR) and identifies the major information items that are relevant for the full documentation and validation of the SLR. It includes administrative and general information entries, methods items, and process indicators. This framework promotes transparency and accountability to SLR registered at the PROSPERO website, which is optional for the Treatabolome contributors.

**Fig. 4:**
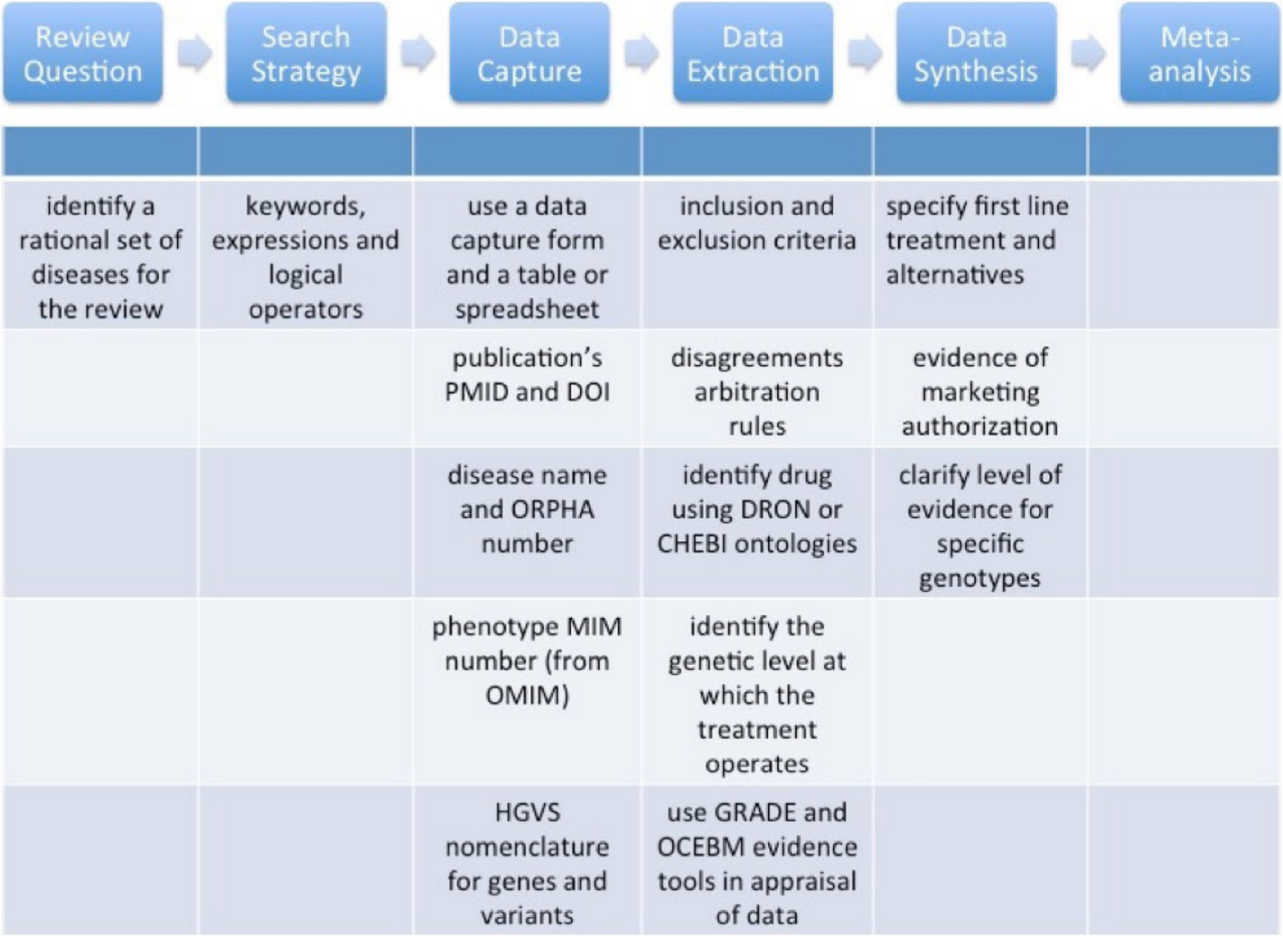
Systematic Review phases: the processes described in the checklists are here expressed in their timeline allocation for the different phases of the Treatabolome Systematic Review process. ChEBI Chemical Entities of Biological Interest. DRON – Drug ontology mapped to RxNorm and ChEBI. GRADE - Grading of Recommendations, Assessment, Development and Evaluations is a transparent framework for developing and presenting summaries of evidence and provides a systematic approach for making clinical practice recommendations. HGVS – Human Genome Variation Society. HPO – Human Phenotype Ontology. OCEBM – Oxford Clinical Eviodence-Based Medicine levels. OMIM - Online Mendelian Inheritance in Man (omim.org). ORDO – Orphanet Rare Diseases Ontology

Figure 4 depicts the details of some of the protocol steps and how they articulate with the different phases of the Systematic Review. A more detailed description can also be found below.

### Systematic review research question

Having found a disease or set of diseases on which to dedicate the systematic literature review (SLR), the next step consists of elaborating the systematic review research question, which is the starting point for any SLR that seeks an answer by identifying all relevant studies, performing quality of evidence appraisals and summarising quantitatively or qualitatively the evidence. We have elaborated a common research question to serve all the tretabolome systematic reviews:

**“What treatments have been described for this condition/gene/variant; on which specific genetic variants have they been tested; and what is the strength of the associated supporting evidence?”**

The question does not address only general treatment considerations but, more specifically, looks for treatments tested for specific genetic causes, which allows to establish gene and variant-specific treatment reports. Whenever this is not possible, other treatment-meaningful genotype-phenotype associations are captured through the integration of the Treatabolome database with other existing resources.

### Search strategy

There is a need to build a search strategy and choose the databases/sources to be inquired to find the answers for the research question. Some examples of databases commonly employed are:

- PubMed
- The Cochrane Central Register of Controlled Trials (CENTRAL)
- ClinicalTrials.gov
- WHO International Clinical Trials Registry Platform (https://www.who.int/ictrp/en/)
- European Clinical Trials Database (EudraCT) https://eudract.ema.europa.eu

The usual number of searched databases is between 1 to 5. The detailed documentation of the search process requires the production of a Search Log to enable replication of the Search Strategy. A Search Log is a document that indicates the date of search, the database or databases queried, the precise search terms and expressions employed, and the number of results returned. Examples of search logs are available online [13], some in spreadsheet format [14], and Additional file 2 contains a Search Log template for exemplification.

Once the list of publications is gathered through the searches reported above, it is advisable to import the results into a bibliography software package that will assist with the exclusion of duplicated references, an essential step before starting the data capture phase. One can also export the list into a spreadsheet and exclude duplicated entries in the spreadsheet software. The spreadsheet list is commonly called the journal citation report and collates the information extracted from the bibliography or reference management software organised by fields. This tool is useful to document the rating of papers according to the inclusion and exclusion criteria, evidence level and other, and provides the accounting for papers excluded and included in the review.

### Data capture

The selected references need to be reviewed, initially by title and abstract analysis followed by full-test analysis whenever necessary. A data capture form based on a Cochrane Collaboration Template is supplied to document the application of inclusion and exclusion criteria and the selection of papers (Additional file 3). When compared to the spreadsheet headings in Table 1, it appears that the data capture form is much more complex, which implies the need to tailor it to the specific needs of the selected disease groups instead of using it regardless. The Treatabolome systematic reviews have broad Inclusion criteria that tolerate all kinds of research, as investigation in rare diseases is usually scarce. The systematic reviews need to accommodate everything from case reports of treatment effects up to double-blind randomised controlled trials, but that inclusiveness has to be tempered by the careful and expert appraisal of the evidence level of the collected data. It is a fact that patients and relatives have long waited for therapies to arrive. This may generate unrealistic expectations about any emerging treatment and requires careful management by clinicians. The assessment of the level of evidence supporting each treatment assists clinicians in explaining what realistic outcomes can be expected from treatments.

**Table 1:** Codebook specifying data capture form spreadsheet headings

Item	Variable name	Value label
Identification of publication	**pmid**	PubMed Identifier is a unique integer value assigned to each PubMed abstract record (to be distinguished from PMCID that corresponds to full-text records in PMC)
Identification of publication	**doi**	The Digital Object Identifier from the International DOI Foundation is a generic framework for managing identification of content over digital networks.
Identification of publication	**harvard**	Use a full reference in Harvard style as described in https://www.mendeley.com/guides/harvard-citation-guide. Include a full url to the journal homepage each time a DOI and PMID do not exist by searching the url on the NLM catalog online (https://www.ncbi.nlm.nih.gov/nlmcatalog)
Year of publication	**year**	
Authors	**authors**	
Title of Publication	**title**	
Journal of publication	**journal**	
Full abstract	**abstract**	
Phenotype of a group of rare diseases	**gener_pheno**	Rare diseases phenotypical grouping, e.g. Congenital Myasthenic Syndromes CMS
Specific phenotype unde the generic phenotype	**specific_phenotype**	e.g. DOK7-related CMS form under CMS
Type of study	**study_type**	case, series, cohort, non-controlled trial, randomised controlled trial, double blind randomised controlled trial, other
Number of patients	**patients_num**	
Gene	**gene**	
Subtype descriptive name	**subtype_name**	
Subtype OMIM code	**subtype_OMIM**	
Variants	**variants**	
Inheritance and zygoty	**inher_zigoty**	
Drug	**drug1**	
Effect	**effect1**	
Drug	**drug2**	
Effect	**effect2**	
Drug	**drug3**	
Effect	**effect3**	
Contraindications	**contraind**	

#### Inclusion criteria

Peer-reviewed papers in english language, reporting treatment at gene or gene variant levels, no matter the dimension of the population (case, series, cohort or other study population), the timeline of the study (retrospective or prospective), the existence of comparator (no comparator, compared against natural history, cohort study, matched control group), with or without randomisations and with or without blinding. An accurate description of all the characteristics of the study, namely the processes of randomisation and blinding, when existing, assists the accurate appraisal of evidence. Outcomes, qualitative or quantitative, should be mentioned with the intervention.

#### Exclusion criteria

Cases without therapeutic intervention or regarding generic therapeutic interventions (e.g, the benefit of physiotherapy for a diverse group of patients) should be excluded. Studies that do not report outcomes should only be discussed in the narrative summary but not counted as evidence for the systematic review.

The data capture form identifies the publication without ambiguity resorting to DOI and PMID pointers and using Harvard style citations of the paper. Cases without DOI or PMID have to include a URL address collected from the NLM online catalogue (https://www.ncbi.nlm.nih.gov/nlmcatalog) that indicates the web address of the publication’s journal. Further instructions on how to format the bibliographic entries according to Harvard format can be retrieved online (https://www.mendeley.com/guides/harvard-citation-guide), although it may be easier to import all the publications into a bibliography software that formats the references into Harvard mode.

A title and abstract scan allow to select the papers for a full-text review. Papers with specific treatments for identified genes or variants of genes are the main feeders of these systematic reviews. Studies with the selected sensible target disease(s) are to be searched for an indication of gene and variant information and data on inheritance and zygosity before recording the reportedly used drugs, their effects, adverse events or contraindications. Excluded papers should have the exclusion reason documented in a field. The annotations of the papers selected for inclusion include mention to the type of study described, the number of patients involved, the existence of a control group, randomisation and blinding, documenting the implementation of the latter. A common requirement for all the systematic reviews is that the selection and review of papers are graphically depicted using a PRISMA flowchart [15]. A template is supplied in Additional file 4 and further clarification on its use can be found on the PRISMA website (http://prisma-statement.org/PRISMAStatement/FlowDiagram.aspx).

The PRISMA flowchart depicts the number of papers that were dealt with during the Identification, Screening, Eligibility and Inclusion phases of the review process.

The existence of the documentation mentioned above determines the trustworthiness of the systematic reviews by building an audit trail that enables replication of results.

### Data extraction

Data extraction aggregates data in meaningful bundles for analysis, guided by the protocol’s inclusion and exclusion criteria. The process follows the data capture form carefully and later transferred to a data capture table built on the template codebook in Table 1. Most of the time the populations of interest are extractable based on phenotypic and genetic characterisation, treatment employed and observed effects. However, whenever doubts and divergences emerge regarding inclusion and exclusion of research papers, the arbitration mechanism has to be described at length in the protocol, naming who are the reviewers and who is designated to arbitrate in cases where reviewers’ unanimity cannot be reached.

All treatments tried for a specific gene variant need as detailed supporting evidence as possible regarding effects, adverse events and contraindications. In cases where treatment is a pharmacological drug, its unequivocal identification requires clarification by adopting ChEBI nomenclature (https://www.ebi.ac.uk/chebi/init.do). A systematic review frequently returns results of multiple different treatment options, some useful under different circumstances, others ineffective or harmful. All of these possibilities should be captured in the output, as they are all of relevance to clinical decision-making. First-line treatment and alternative options should be presented in clear terms when possible. Weight of evidence may favour a specific treatment as first-line therapy, or a particular therapy may generally be used as adjunctive or as an alternative under certain circumstances. It is necessary to differentiate all these options and map them to the corresponding evidence.

Patients with rare diseases are often risk-takers as a consequence of the scarcity of innovation and research in rare disease areas. For patients’ safeguard, the Treatabolome opts for using a transparent and sensible approach for grading the quality of evidence and strength of recommendations in health care. This choice reflects our vision that is centred on the clinical significance of the findings and not exclusively on the statistical significance or effects size. We believe that is the right focus for establishing a health technology assessment for these treatments in a hopefully near future.

We adopted the GRADE methodology for appraising the evidence collected and reaching the Treatabolome database treatment recommendations. The GRADE questionnaire is part of the Data Capture form supplied and is also available online through a resource that is free for academic institutions, the GRADE Pro website (https://gradepro.org). An online handbook and other resources are available at the website to support users (https://gdt.gradepro.org/app/handbook/handbook.html).

The Oxford Centre for Evidence-Based Medicine has issued Levels of Evidence to assist in the appraisal of published scientific evidence (http://www.cebm.net/index.aspx?o=5653). This helpful tool allows for a quick overview of data quality and its parallel use reinforces the objectivity of the Treatabolome recommendations. Other tools can also be considered like the Jadad Score [16], although the latter is applied for appraising clinical trials and these are scarce in the rare diseases’ field.

### Data synthesis

The synthesis phase follows the extraction phase, and it addresses the quantitative and qualitative aspects separately. The qualitative analysis employs different tools depending on the objectives of the systematic review [17]:

- narrative analysis extracts key concepts from a study
- narrative summary does a textual combination of data, namely when qualitative studies are heterogeneous (meta-synthesis)
- meta-ethnography synthesises the qualitative data to reach new theoretical understandings.

The synthesis of quantitative data differs depending on the heterogeneity of studies. The meta-analysis, an operation that combines data from different studies, should only be performed if data from intervention studies are similar, and it is sensible to combine the studies. When it is feasible, meta-analysis enhances the degree of certainty when answering a research question as it boosts the power of data by aggregation of datasets [18]. Details about the sophisticated meta-analysis methodology are beyond this paper’s scope and so the reader is referred to existing quality resources if necessary [19, 20].

The majority of rare diseases’ treatment publications are single case or case series-reports, having a low-evidence level, high heterogeneity of methods, and frequently using no comparators or only historical ones. On the other hand, as the objective is to identify gene and variant-specific treatment reports it is harder to identify homogeneous studies on which to perform a meta-analysis, as studies are both scarce and heterogeneous regarding populations, methodologies or interventions. Gathering conditions to perform a meta-analysis in this scenery is therefore unlikely. Considering the above limitations, it is fortunate that data synthesis still brings enlightenment by aggregating data in tables, performing a narrative summary and counting the papers reporting a similar effect. The structure of this summary is essential and should be planned. In the current project, the decisions regarding reporting are discussed in the protocol of the systematic review and data synthesis should follow the spreadsheet template provided as codebook in Table 2. Consequently, the authors of the systematic review should try when possible to indicate the first line of treatment and potential alternatives for each genotype and indicate the corresponding level of evidence for each of the treatment modalities. As treatments may already have marketing authorizations, usually for other conditions, details of the authorization should also be captured and discussed in terms of access to therapy and orphan drug designation.

**Table 2:** codebook specifying summary table column headings (variable names)

Item	Variable name	Value label
Phenotype of a group of rare diseases	**gener_pheno**	Rare diseases phenotypical grouping, e.g. Congenital Myasthenic Syndromes CMS
Specific phenotype under the generic phenotype	**spec_pheno**	Rare diseases subtype under a generic phenotype, e.g. DOK7 under CMS
Gene	**gene**	
First-line treatment recommendation	**first_line**	
Alternative treatment 1	**alt_treat_1**	
Alternative treatment 2	**alt_treat_2**	
Likely innefective	**innefective**	List of medications tried without success
Harmful	**harmful**	List of medications that can harm these patients
Expert Summary of Evidence	**expert_sum**	
number of publications	**num_pub**	Integer that corresponds to the total of publications reviewed for this database entry
Key reference	**key_ref**	Main literature reference for the database entry
PMIDs	**pmids**	List the PMIDs of papers referenced for this database entry
DOIs	**dois**	List of DOIs of papers referenced for this database entry
OCEBM	**ocebm**	OCEBM classification of evidence
GRADE	**grade**	GRADE classification of evidence

## Discussion

The Treatabolome concept fills in a missing link in the existing diagnostic support tools, enabling the existing treatments for rare diseases to have maximal visibility to avoid adding treatment delay to the rare disease diagnostic odyssey that often deprives patients and families of diagnosis over a significant time. The Treatabolome project fulfils that objective by liaising with existing diagnostic and support tools and providing treatment information at the gene and variant level whenever possible. Immediate treatment visibility at the moment of diagnosis is the primary goal of the project and to trigger it full Systematic Literature Reviews must supply the expert-reviewed information. The continuous update of this information requires recurrent scanning of the literature that may in the future depend on text-mining of scientific papers instead of repeated expert-led literature reviews. The experts that are part of the four participating European Reference Networks of the Solve-RD European Project were invited to cooperate on the Treatabolome tasks ahead of others. Still, the natural evolution of the task presupposes the growing involvement of a multitude of experts across the world.

## Conclusions

In general, systematic reviews are still the best tool to guarantee that decisions regarding healthcare and research are guided by the best existing evidence to provide the best possible care to patients. Despite the efforts done for automation of systematic reviews [21, 22], the process is still mostly dependent on human effort, and the Treatabolome ones are characterised by the extra challenge of trying to find gene and variant-specific treatments while appraising their level of evidence through consecrated tools, as the GRADE system and the Oxford Evidence-Based Medicine Levels. It is our hope that the current paper assists researchers in developing Treatabolome systematic reviews and producing datasets for future incorporation in the Treatabolome knowledge base and diagnostic support tools.

## Supplementary information

**Additional file 1.** Systematic Review Protocol.

**Additional file 2.** Search Log.

**Additional file 3.** Data Capture Form.

**Additional file 4.** PRISMA Flow Diagram.

## Data Availability

Data sharing is not applicable to this article as no datasets were generated or analysed during the current study.

## Abbreviations

BD2K: Big data to knowledge
CENTRAL: Cochrane Central Register of Controlled Trials (https://www.cochranelibrary.com/central/about-central)
ChEBI: Chemical Entities of Biological Interest (ChEBI) is a freely available dictionary of molecular entities focused on ‘small’ chemical compounds (https://www.ebi.ac.uk/chebi/init.do)
DOI: Digital object identifier (doi.org)
ERN: European Reference Networks
EudraCT: European Clinical Trials Database (https://eudract.ema.europa.eu)
FAIR: The FAIR guiding principles of data management and stewardship
IRDiRC: International Rare Diseases Research Consortium (www.irdirc.org)
NLM: National Library of Medicine
PMID: Unique identifier number used in PubMed
PubMed: Resource supporting the search and retrieval of peer-reviewed biomedical and life sciences literature (https://www.ncbi.nlm.nih.gov/pubmed/)
PRISMA: Preferred Reporting Items for Systematic Reviews and Meta-Analyses (http://prisma-statement.org/Default.aspx)
PROSPERO: International prospective register of systematic reviews (https://www.crd.york.ac.uk/PROSPERO/)
Solve-RD: Solve-RD - solving the unsolved rare diseases (solve-rd.eu)
SLR: Systematic Literature Review
UDNI: Undiagnosed Diseases Network International

## References

[1] Dawkins HJS, Draghia-Akli R, Lasko P, Lau LPL, Jonker AH, Cutillo CM (2018). Progress in rare diseases research 2010-2016: an IRDiRC perspective. Clin Transl Sci.

[2] Nguengang Wakap S, Lambert DM, Olry A, Rodwell C, Gueydan C, Lanneau V (2019). Estimating cumulative point prevalence of rare diseases: analysis of the Orphanet database. Eur J Hum Genet.

[3] Kempf L, Goldsmith JC, Temple R (2018). Challenges of developing and conducting clinical trials in rare disorders. Am J Med Genet A.

[4] Swan_UK W, Eurordis, Rare_Voices_Australia, CORD, NORD, ASRID (2016). International joint recommendations to address specific needs of undiagnosed rare disease patients.

[5] Boycott KM, Campeau PM, Howley HE, Pavlidis P, Rogic S, Oriel C (2020). The Canadian rare diseases models and mechanisms (RDMM) network: connecting understudied genes to model organisms. Am J Hum Genet.

[6] Thompson R, Bonne G, Missier P, Lochmuller H (2019). Targeted therapies for congenital myasthenic syndromes: systematic review and steps towards a treatabolome. Emerg Top Life Sci.

[7] Austin CP, Cutillo CM, Lau LPL, Jonker AH, Rath A, Julkowska D (2018). Future of rare diseases research 2017-2027: an IRDiRC perspective. Clin Transl Sci.

[8] Southall NT, Natarajan M, Lau LPL, Jonker AH, Deprez B, Guilliams T (2019). The use or generation of biomedical data and existing medicines to discover and establish new treatments for patients with rare diseases - recommendations of the IRDiRC data mining and repurposing task force. Orphanet J Rare Dis.

[9] EU (2017). European reference networks.

[10] Pletscher-Frankild S, Palleja A, Tsafou K, Binder JX, Jensen LJ (2015). DISEASES: text mining and data integration of disease-gene associations. Methods..

[11] Wilkinson MD, Dumontier M, Aalbersberg IJ, Appleton G, Axton M, Baak A (2016). The FAIR guiding principles for scientific data management and stewardship. Sci Data.

[12] CRD (2019). PROSPERO - international register of systematic reviews.

[13] UoSC (2019). Searching solutions: the search log.

[14] University_of_Tasmania (2019). Systematic reviews for health: documenting search strategies.

[15] PRISMA (2019). Transparent reporting of systematic reviews and meta-analyses.

[16] Jadad AR, Moore RA, Carroll D, Jenkinson C, Reynolds DJ, Gavaghan DJ (1996). Assessing the quality of reports of randomized clinical trials: is blinding necessary?. Control Clin Trials.

[17] Munn ZT CA, E. Data extraction and synthesis. The steps following study selection in a systematic review. [Available from: https://alliedhealth.ceconnection.com/files/DataExtractionandSynthesis-1430416358306.pdf.

[18] Haidich AB (2010). Meta-analysis in medical research. Hippokratia..

[19] Borenstein M (2009). Introduction to meta-analysis.

[20] Cochrane_Collaboration. Collaboration C (2011). Cochrane handbook for systematic reviews of interventions version 5.1.

[21] Marshall IJ, Wallace BC (2019). Toward systematic review automation: a practical guide to using machine learning tools in research synthesis. Syst Rev.

[22] van Altena AJ, Spijker R, Olabarriaga SD (2019). Usage of automation tools in systematic reviews. Res Synth Methods.

