# Correction to: A guide to writing systematic reviews of rare disease treatments to generate FAIRcompliant datasets: building a Treatabolome

**DOI:** 10.1186/s13023-021-01777-6

**Published:** 2021-03-22

**Authors:** Antonio Atalaia, Rachel Thompson, Alberto Corvo, Leigh Carmody, Davide Piscia, Leslie Matalonga, Alfons Macaya, Angela Lochmuller, Bertrand Fontaine, Birte Zurek, Carles Hernandez-Ferrer, Carola Reinhard, David Gómez-Andrés, Jean-François Desaphy, Katherine Schon, Katja Lohmann, Matthew J. Jennings, Matthis Synofzik, Olaf Riess, Rabah Ben Yaou, Teresinha Evangelista, Thiloka Ratnaike, Virginie Bros-Facer, Gulcin Gumus, Rita Horvath, Patrick Chinnery, Steven Laurie, Holm Graessner, Peter Robinson, Hanns Lochmuller, Sergi Beltran, Gisèle Bonne

**Affiliations:** 1 Sorbonne Universite - Inserm UMRS 974, Center of Research in Myology, Institut de Myologie, G.H. Pitié-Salpêtrière Paris, 47, boulevard de l’Hopital, 75 651 Paris Cedex 13, France; 2 Children’s Hospital of Eastern Ontario Research Institute, Ottawa, Canada; 3 CNAG-CRG, Centre for Genomic Regulation (CRG), Barcelona Institute of Science and Technology (BIST), Barcelona, Spain; 4 The Jackson Laboratory for Genomic Medicine, Farmington, CT 06032 USA; 5 Paediatric Neurology, Vall D’Hebron University Hospital and VHIR (Euro-NMD, ERN-RND), 08035 Barcelona, Spain; 6 Department of Clinical Neurosciences, University of Cambridge School of Clinical Medicine, Cambridge Biomedical Campus, Cambridge, UK; 7 Institute of Medical Genetics and Applied Genomics, University of Tuebingen, Tübingen, Germany; 8 Department of Biomedical Sciences and Human Oncology, School of Medicine, University of Bari Aldo Moro, Bari, Italy; 9 Institute of Neurogenetics, University of Lübeck, 23538 Lübeck, Germany; 10 Department of Neurodegenerative Diseases, Hertie-Institute for Clinical Brain Research and Center of Neurology, University of Tübingen, Tübingen, Germany; 11 German Center for Neurodegenerative Diseases (DZNE), University of Tübingen, Tübingen, Germany; 12 Unité de Morphologie Neuromusculaire, Institut de Myologie, GHU Pitié-Salpêtrière, Paris, France; 13 Sorbonne Université, AP-HP, INSERM, Centre de Référence Des Maladies Neuromusculaires Nord/Est, Ile de France, Paris, France; 14 Department of Paediatrics, Cambridge University Hospitals NHS Foundation Trust, Cambridge, England; 15 Department of Clinical Neurosciences, University of Cambridge, Cambridge, UK; 16 EURORDIS – Rare Diseases Europe, Paris, France; 17 Department of Clinical Neurosciences, School of Clinical Medicine, University of Cambridge and MRC Mitochondrial Biology Unit, Cambridge Biomedical Campus, Cambridge, UK; 18 Department of Neuropediatrics and Muscle Disorders, Medical Center - University of Freiburg, Faculty of Medicine, University of Freiburg, Freiburg im Breisgau, Germany; 19 Division of Neurology, Department of Medicine, The Ottawa Hospital, Ottawa, Canada; 20 Brain and Mind Research Institute, University of Ottawa, Ottawa, Canada

## Correction to: *Orphanet J Rare Dis* (2020) 15:206 https://doi.org/10.1186/s13023-020-01493-7

The original version of this article [1] unfortunately included a typo to a co-author’s name.

Author Carola Reinhard was erroneously presented as Carola Rheinard.

The correct author name is shown in the author list of this Correction and has already been updated in the original article.
