# Supplementary material for: A guide to writing systematic reviews of rare disease treatments to generate FAIR-compliant datasets: building a Treatabolome
Source: Orphanet J Rare Dis. 2020 Aug 12;15:206. doi: 10.1186/s13023-020-01493-7 (PMC7424983; doi:10.1186/s13023-020-01493-7)
Supplement: Supplementary file 1 — Additional file 1. Systematic Review Protocol. [file 13023_2020_1493_MOESM1_ESM.docx]

Annex I

Systematic Review Protocol

ANNEX I

ANNEX I

***Systematic review of treatments reported for [CONDITION]***

[authors list]

Citation

… Systematic review of treatments reported for [CONDITION]. PROSPERO 2019 [Prospero number] Available from: url

Systematic Review Research Question

“What treatments have been described for this condition; on which specific genetic causes have they been tested; and what is the strength of the associated supporting evidence?”

Searches

The following databases will be searched:

 …

 …

 …

Types of study to be included

All clinical trials, randomised or not, blinded or not, quasi-experimental/CCT and pre-experimental studies about any modality of treatment, pharmacological, genetic, physical, device-related used in [CONDITION] patients. The trials may have a control or comparison group or not or being assessed against a natural history cohort or not. Due to scarcity of data for rare diseases in general, cohort, series and case studies are considered even when only qualitative outcomes are available.

Condition or domain being studied

Describe [CONDITION]

Participants/population

All published reports, primary research papers or other publicly available data available for review identified according to the above-described research strategy are to be assessed against our inclusion and exclusion criteria, independently of presenting the results of clinical trials (randomised or not, blinded or not, controlled or not), cohort studies, case series or single case reports. This option is dictated by the scarcity of rare diseases' research and the consequent need to be inclusive of any available evidence. There will possibly be insufficient data for a meta-analysis, but in that case, data synthesis is performed instead. The qualitative outcomes are to be considered in the narrative summary.

Interventions/Exposures

ANNEX I

All kind of interventions is considered for the purpose of the review.

Comparator(s)/control

The comparator may be historical controls, a cohort study group or a control group. Cases series or case reports without comparator are taken into consideration, even when they do not report N-of-1 trials.

Context

All reported patients with a condition diagnosed as arising from a disorder in the [CONDITION] gene or one of its variants.

Main outcome

Any outcome reported will be considered, independently of reporting a quantified improvement of disease signs or symptoms or a biomarker change that is accepted to express clinical improvement.

Additional outcomes

Qualitative improvement reports are considered as well, but only for the effect of writing the narrative summary.

Data extraction (selection and coding)

 **Selection of Studies**

Databases will be searched by the primary researcher (NAMES). The number of citations found will be documented, namely in a PRISMA data flowchart, and a list will be compiled of studies that potentially meet the inclusion criteria (titles and abstracts). After searching all databases, two reviewers (NAMES) will independently review the search results and lists. Following independent selection of papers for full text review, the authors will compare findings and any disagreement will be resolved by discussion in order to reach consensus. Where disagreement cannot be resolved a third peer (NAMES) will be asked to arbitrate.

 **Data extraction and management**

Two reviewers (NAMES) will independently extract data from published reports or original research with a pre-structured data extraction form. Information will be collected on participants (demographic data, diagnosis, clinical picture, inclusion/exclusion criteria, co-morbidities, trial setting, allocation procedure, blinding, number of patients), interventions (pharmacological, genetic, device-related, physical or other non-pharmacological), outcome measures and biomarkers used, results (outcome measures, descriptive statistics, number of participants). Stratification of data according to gene, variant, phenotype, diagnosis and other pertinent variables will be done. When necessary, the correspondent author for the published datasets may be contacted to complete missing information or clear doubts. If any disagreements occur between the two researchers in charge of extracting data, a third reviewer will be called to arbitrate, unless the situation is resolved through discussion and consensus. The primary researcher (NAMES) will enter the data in bibliography management software with the accuracy of data capturing confirmed by another reviewer (NAMES). The bibliography software will execute de-duplication of records and a data capture spreadsheet with the results is generated in accordance with the supplied template.

ANNEX I

Risk of bias (quality) assessment

The methodological quality of the included studies is independently assessed by two reviewers (NAMES) using the Oxford Evidence-Based Medicine Levels and the GRADE system. The following aspects require assessment: selection bias, performance bias, detection bias, attrition bias, reporting bias and others. This procedure aims at identifying appropriate indicators that characterise the quality of the studies. We use two different systems to reinforce the need for transparency and precision to avoid over-optimistic estimates that may risk putting patients at harm. Rare disease patients are usually motivated to accept higher risks because of the scarcity of treatments, and therefore we need reinforced caution and clarity when explaining treatment estimates, side effects or odds of coming to harm to them.

Strategy for data synthesis

The level of heterogeneity of the studies need assessment and if necessary statistical tools are employed to bring up the evidence objectively. In the latter case, a Chi-square of p<0.1is considered statistically significant. If there are a sufficient number of included studies in the final sample, then a funnel plot helps to determine reporting bias. However, most of the times, this may not be possible to achieve due to data scarcity. If a meta-analysis cannot be performed for whatever reason (scarcity of data, data heterogeneity), then a narrative synthesis replaces it. A table summary template in compliance with the FAIR guiding principles of data management can be found in an attachment to this protocol. Missing data problems are whenever possible to be solved contacting the corresponding authors. Intention-to-treat analysis is the recommended method to deal with missing data from drop-out study participants.

Analysis of subgroups or subsets

There will be subgroups analysis organised for each clinically meaningful phenotypical grouping, as discussed above.

Contact details

Corresponding author:

Organizational affiliation of the review

Review team members and their organisation affiliations

Type and method of review

ANNEX I

Anticipated or actual start date

Anticipated completion date

Funding sources or sponsors

Conflicts of interest

Language

English

Country

Stage of review

Subject index terms status

Subject index terms

Date of registration in PROSPERO

Date of publication of this version

Details of any existing review of the same topic by the same authors

Stage of review at time of this submission
