# Supplementary material for: A guide to writing systematic reviews of rare disease treatments to generate FAIR-compliant datasets: building a Treatabolome
Source: Orphanet J Rare Dis. 2020 Aug 12;15:206. doi: 10.1186/s13023-020-01493-7 (PMC7424983; doi:10.1186/s13023-020-01493-7)
Supplement: Supplementary file 2 — Additional file 2. Search Log. [file 13023_2020_1493_MOESM2_ESM.docx]

Annex II

Search Log

| Date | Database/Source^[[1]](#footnote-1)^ | Language | Search Expression(s)^[[2]](#footnote-2)^ | Results returned |
| --- | --- | --- | --- | --- |
|  |  |  |  |  |
|  |  |  |  |  |
|  |  |  |  |  |

1. the names of the databases you searched or the sources explored, like grey literature (e.g. conference proceedings) and websites [↑](#footnote-ref-1)
2. Including filters like date ranges or publication type (e.g. clinical trials only) [↑](#footnote-ref-2)
