# Supplementary material for: A guide to writing systematic reviews of rare disease treatments to generate FAIR-compliant datasets: building a Treatabolome
Source: Orphanet J Rare Dis. 2020 Aug 12;15:206. doi: 10.1186/s13023-020-01493-7 (PMC7424983; doi:10.1186/s13023-020-01493-7)
Supplement: Supplementary file 3 — Additional file 3. Data Capture Form. [file 13023_2020_1493_MOESM3_ESM.docx]

Annex III

Data Capture Form

Available online from Cochrane website:

<https://www.google.com/url?sa=t&rct=j&q=&esrc=s&source=web&cd=2&ved=2ahUKEwisu5_DybvjAhWM2BQKHXduDlcQFjABegQIARAC&url=https%3A%2F%2Fcommunity.cochrane.org%2Fsites%2Fdefault%2Ffiles%2Fuploads%2Finline-files%2FERC%2520data%2520collection%2520form%2520for%2520intervention%2520reviews%2520for%2520RCTs%2520and%2520non-RCTs.doc&usg=AOvVaw2f0Wx5fM1jBDFLsLBbNpI->

ANNEX III
