# Supplementary material for: A guide to writing systematic reviews of rare disease treatments to generate FAIR-compliant datasets: building a Treatabolome
Source: Orphanet J Rare Dis. 2020 Aug 12;15:206. doi: 10.1186/s13023-020-01493-7 (PMC7424983; doi:10.1186/s13023-020-01493-7)
Supplement: Supplementary file 4 — Additional file 4. PRISMA Flow Diagram. [file 13023_2020_1493_MOESM4_ESM.docx]

**
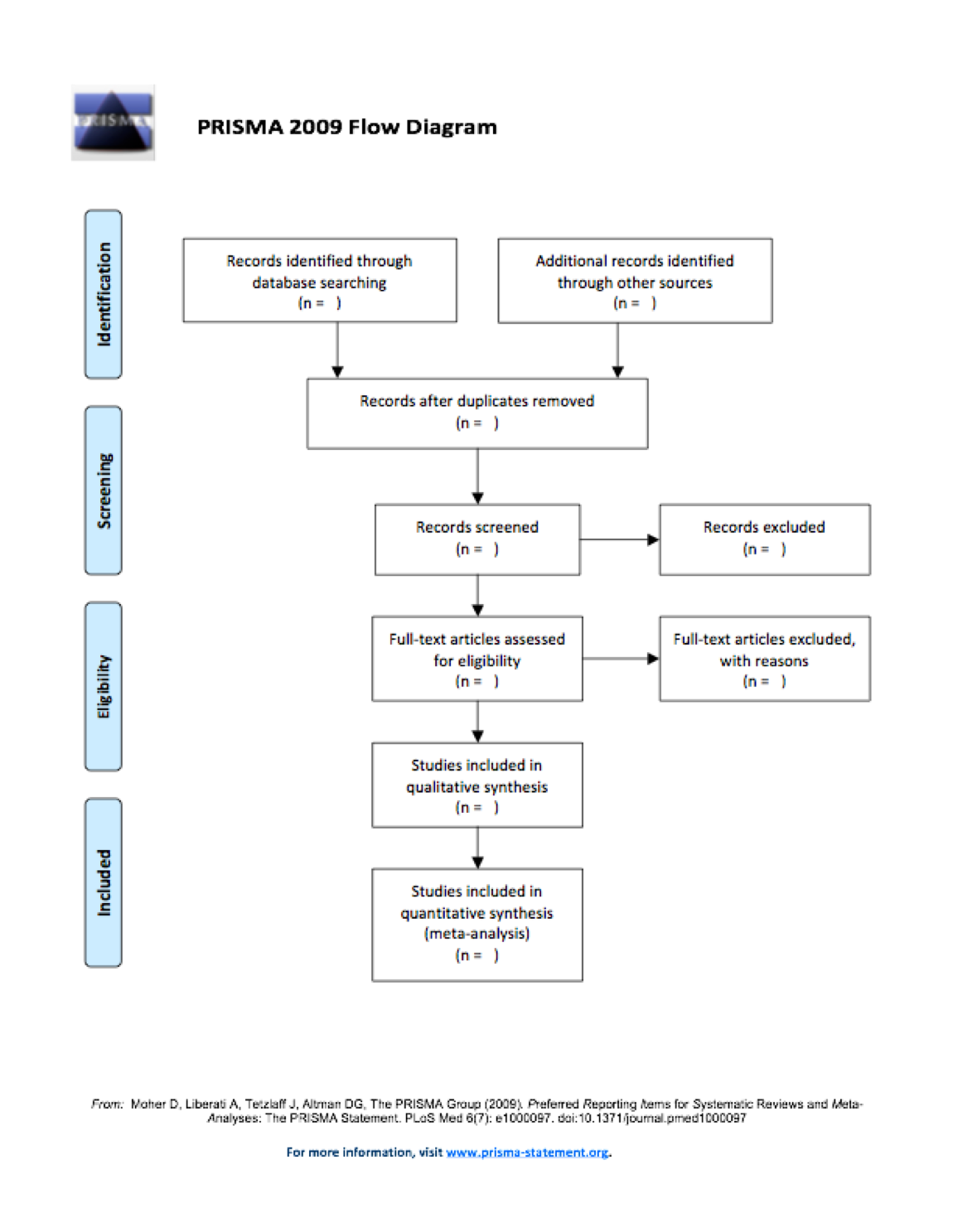
**

**TABLE OF CONTENTS**

| SUBJECT | PAGE |
| --- | --- |
| Abstract | 2 |
| Background | 3 |
| Aims | 5 |
| Methods | 6 |
| Systematic Review Protocol | 6 |
| Systematic Review Research Question | 6 |
| Search Strategy | 7 |
| Data Capture | 8 |
| Data Extraction | 9 |
| Data Synthesis | 11 |
| Discussion | 12 |
| Conclusions | 12 |
| List of Abbreviations | 13 |
| Acknowledgments | 14 |
| Funding | 14 |
| Competing Interests | 14 |
| Authors’ Contributions | 14 |
| Availability of Data and Materials | 14 |
| Ethicals approval and consent to participate | 15 |
| Consent for publication | 15 |
| Bibliography | 16 |
| Figure 1 – The Treatabolome Overview | 18 |
| Figure 2 – The Treatabolome is a precision medicine project | 19 |
| Figure 3 – The Treatabolome Systematic Review Checklist | 20 |
| Figure 3 – Systematic Review Phases | 21 |
| Table I - codebook specifying data capture form spreadsheet headings | 22 |
| Table II - codebook specifying summary table column headings (variable names) | 23 |
| Annex I - Systematic Review Protocol | 24 |
| Annex II - Search Log | 29 |
| Annex III - Data Capture Form | 31 |
| Annex IV - PRISMA Flow Diagram | 33 |
